# Evaluation of Signature Erosion in Ebola Virus Due to Genomic Drift and Its Impact on the Performance of Diagnostic Assays

**DOI:** 10.3390/v7062763

**Published:** 2015-06-17

**Authors:** Shanmuga Sozhamannan, Mitchell Y. Holland, Adrienne T. Hall, Daniel A. Negrón, Mychal Ivancich, Jeffrey W. Koehler, Timothy D. Minogue, Catherine E. Campbell, Walter J. Berger, George W. Christopher, Bruce G. Goodwin, Michael A. Smith

**Affiliations:** 1 Critical Reagents Program, Medical Countermeasure Systems Annex, 110 Thomas Johnson Drive, Frederick, MD 21702, USA; E-Mails: bruce.g.goodwin4.civ@mail.mil (B.G.G.); michael.a.smith215.civ@mail.mil (M.A.S.); 2 The Tauri Group, LLC, Alexandria, VA 22310, USA; 3 Noblis, Inc., 3150 Fairview Park Drive South, Falls Church, VA 22042, USA; E-Mails: mitchell.holland@noblis.org (M.Y.H.); daniel.negron@noblis.org (D.A.N.); mychal.ivancich@noblis.org (M.I.); walter.berger@noblis.org (W.J.B.); 4 Diagnostic Systems Division, United States Army Medical Research Institute of Infectious Diseases, Fort Detrick, MD 21702, USA; E-Mails: adrienne.t.hall2.civ@mail.mil (A.T.H.); jeff.w.koehler.ctr@mail.mil (J.W.K.); timothy.d.minogue.civ@mail.mil (T.D.M.); 5 DCE consulting, Vienna, VA 22181, USA; E-Mail: dceconsulting@verizon.net; 6 Medical Countermeasure Systems, Ft. Belvoir, VI 22060, USA; E-Mail: george.w.christopher.civ@mail.mil

**Keywords:** EBOV, Western African outbreak, WGS, qRT-PCR, signature erosion, PSET, Bio*Velocity*

## Abstract

Genome sequence analyses of the 2014 Ebola Virus (EBOV) isolates revealed a potential problem with the diagnostic assays currently in use; *i.e.*, drifting genomic profiles of the virus may affect the sensitivity or even produce false-negative results. We evaluated signature erosion in ebolavirus molecular assays using an *in silico* approach and found frequent potential false-negative and false-positive results. We further empirically evaluated many EBOV assays, under real time PCR conditions using EBOV Kikwit (1995) and Makona (2014) RNA templates. These results revealed differences in performance between assays but were comparable between the old and new EBOV templates. Using a whole genome approach and a novel algorithm, termed Bio*Velocity*, we identified new signatures that are unique to each of EBOV, Sudan virus (SUDV), and Reston virus (RESTV). Interestingly, many of the current assay signatures do not fall within these regions, indicating a potential drawback in the past assay design strategies. The new signatures identified in this study may be evaluated with real-time reverse transcription PCR (rRT-PCR) assay development and validation. In addition, we discuss regulatory implications and timely availability to impact a rapidly evolving outbreak using existing but perhaps less than optimal assays *versus* redesign these assays for addressing genomic changes.

## 1. Introduction

Ebola virus disease (EVD) is one of the deadliest infectious diseases that has ravaged Central and Western Africa many times in the past, and has evoked global fear while testing the global community’s preparedness and response during the ongoing 2014 Western African epidemic [1]. This is in part due to the high case-fatality ratio (CFR) of EVD, the general lack of control measures in resource-poor nations to curtail transmission, and a lack of approved medical countermeasures such as vaccines or antiviral therapeutics. The genus *Ebolavirus* has five members with genetic diversity ranging from 25%–35%: Tai Forest virus (TAFV), Reston virus (RESTV), Sudan virus (SUDV), Ebola virus (EBOV), and Bundibugyo virus (BDBV) [2]. There have been at least 41 filovirus disease outbreaks in humans during the period between 1967 and 18 September 2014. In addition, ebolavirus has emerged repeatedly in animal populations in Central and Western Africa, causing massive die-offs of gorillas and chimpanzees [3]. The CFRs in major human outbreaks vary from 25% to 90% depending on the viral species, number of cases and other factors, such as access to healthcare [1]. 

The ongoing EVD epidemic originated in Guinea in December 2013 and is the largest in recorded history, affecting multiple countries in Western Africa [4]. According to an Ebola Response model constructed by the U.S. Centers for Disease Control and Prevention (CDC), if trends had continued without scale-up of effective interventions or changes in community behavior (e.g., early diagnosis and hospitalization in Ebola Treatment Units and reductions in unsafe burial practices), it was estimated that there would have been as many as 1.4 million cases by 20 January 2015 [5]. Revised estimates by a different group predicted a similar trend in the absence of effective steps to curtail transmission [6]. However, as of January 2015, the trend appears to have slowed as opposed to the exponential increase predicted by the model, despite the unprecedented epidemiological scale of this outbreak. The limited success in containment can be attributed to the effectiveness of some of the control measures put in place in affected countries [7]. As of 17 May 2015 there have been 26,969 total cases (suspected, probable, and confirmed), 14,991 laboratory-confirmed cases, and 11,135 total deaths amounting to a case fatality ratio (CFR) of 41.2% in Western Africa, and a few imported and secondary cases in various other countries including the U.S [8].

A recent study has suggested that the ongoing epidemic in Western Africa originated from a single zoonotic transmission event from a free-tailed bat (*Mops condylurus*) to a two-year-old boy in Meliandou, Guinea, who is believed to have been the index case [9]. Hence, active environmental biosurveillance can help predict/prevent the transmission of the virus from its natural reservoirs to humans [10]. In addition, rapid, sensitive, safe and simple diagnostic tests may expedite case finding and the timely initiation of medical and outbreak control measures to interrupt viral transmission in resource constrained settings, such as Ebola Treatment Units. Such a measure can also help in preventing nosocomial infections, guiding triage and clinical decisions, aiding contact tracing and facilitating the early isolation of cases [11]. Efforts to contain the EVD outbreak in Western Africa are currently hampered by cumbersome, slow, complex, and expensive diagnostic tests that impose a number of additional logistical challenges, including requirements for a high level of laboratory biosafety and staff expertise in using sophisticated instruments [11].

Hence, many laboratories and private organizations are developing and deploying handheld tests for rapid detection of EBOV in clinical specimens to address this gap [12]. Recently, Corgenix Medical Corporation received an Emergency Use Authorization (EUA) approval from the U.S. Food and Drug Administration (FDA) for its ReEBOV™ Antigen Rapid Test. In addition, several laboratory-developed immunoassays for different platforms (e.g., ELISA, MAGPIX) are currently in use for EBOV testing. 

The current gold standard for EBOV detection is rRT-PCR molecular assays that can be run on various PCR instruments [(Applied Biosystems real time PCR systems, Joint Biological Agent Identification and Diagnostic System (JBAIDS), Light Cycler *etc.*)] for real time detection of EBOV from serum and plasma. Several organizations, including the U.S. Department of Defense (DoD), have deployed detection kits based on PCR assays. Early PCR testing enhances the medical care of both infected and uninfected patients, and guides rational hospital infection control. Real-time RT-PCR tests are also valuable for correlating viral load to disease severity. Rapid detection tests can rule out non-EVD cases if other PCR assays are available to test for the etiologic agents of diseases that present with similar symptoms. Expanded PCR testing would also enable detecting co-infection in EVD patients and guide treatments accordingly. Additionally, they can be used for post-infection tracking of asymptomatic shedding in convalescent patients including documentation of virus in the semen and breast milk weeks after resolution of symptoms [13]. 

The FDA has, thus far, approved seven molecular assays through the Emergency Use Authorization (EUA) process, and these include [14]: (1) CDC Ebola Virus VP40 Real-time RT-PCR Assay; (2) CDC Ebola Virus NP Real-time RT-PCR Assay; (3) DoD EZ1 Real-time RT-PCR Assay based on VP40; (4) BioFire Defense LLC FilmArray NGDS BT-E Assay; (5) BioFire Defense LLC FilmArray Biothreat-E Test; (6) RealStar^®^ Ebolavirus RT-PCR Kit 1.0; and (7) LightMix^®^ Ebola Zaire rRT-PCR Test. Many other assays; e.g., OasigTM-rRT-PCR and the DoD EZ2 assay, both based on the NP gene, are in the EUA pipeline. However, many of the assays were developed using sequence information prior to the 2014 outbreak or from a limited number of sequences obtained early in the current outbreak. The assay signatures of preexisting assays have not been systematically and periodically evaluated against new genome/gene sequences that are deposited in GenBank in order to assess their performance against new viral lineages.

In a landmark genomic study, Gire *et al.* sequenced 99 viral genomes in samples originating from 78 patients early in the 2014 Western African outbreak [15]. Results based on in depth genome sequence analyses revealed a number crucial factors about viral transmission dynamics and genome plasticity of 2014 EBOV: (1) the finding that the potential source of the Western African variant is a central African lineage from 2004; (2) intra- and inter-host genetic variations; (3) sustained human-to-human transmission from the initial jump from a natural reservoir without additional inter host transmission; and (4) very high mutation rates typical of RNA viruses (8 × 10^−4^ per base per year) that approaches the mutational rate of seasonal influenza [16]. This study also highlighted the fact that many of the mutations alter RNA and protein sequences that are targets for diagnostics, vaccines and therapeutics and hence the need to redesign existing diagnostics (and therapeutics in the pipeline) as well as accelerate the development and approval of medical countermeasures undergoing clinical trials [15]. A recent report has assessed the potential impact of genomic changes in EBOV on the efficacy of sequence-based candidate therapeutics [17].

In this work, we have focused on the potential limitations of the ebolavirus molecular assays by assessing (1) signature erosion due to viral genetic changes [the term ‘signature-erosion’ is used here to signify potential false-positive or false-negative results in molecular assays due to mutations in the PCR primers/probe/amplicon target sequences (PCR signatures)] [18,19]; (2) the need for addressing genomic changes when re-optimizing old assays or designing new assays; (3) a critical need for capturing the genetic diversity of ebolavirus from geographically and temporally diverse isolates; and (4) a need for deep sequencing to capture the quasi-species evolution within individuals over time (e.g., longitudinal studies). Rapid generation of sequence data at the onset and throughout the course of an outbreak is critical for establishing confidence in the fielded diagnostic assays and, if required, for the rapid development of new assays or optimization of old assays. Deep sequencing would allow prediction and development of proactive signatures, in the event rare variants present in a quasi-species that have a predictable effect on existing signatures become prevalent. We draw the attention of the research community to newer approaches to molecular assay design that takes a whole genome approach rather than gene-centric approach and long term solutions to assay design that are proactive rather than reactive for new and reemerging threats. We also discuss mitigation strategies and the need for: crowd sourcing of data, analyses and rapid development of assay quality control measures, regulatory approvals and future technologies. There are other assay limitations such as time to result, sensitivity and specificity, and issues with sample collection and preparation that are not directly addressed here.

## 2. Materials and Methods

### 2.1. Viruses and Sequencing

Two different EBOV isolates used in this study were obtained from the Critical Reagents Program [20] (Unified Culture Collection (UCC) [21] These viruses included EBOV/H.sapiens-tc/COD/1995/Kikwit (UCC# R4368) and EBOV/H.sapiens-tc/SLE/2014/Makona (UCC# R4491). Total nucleic acid was purified from 200 µL cell culture supernatant in TRIzol (Life Technologies, Grand Island, NY, USA) using the EZ1 Virus 2.0 kit (Qiagen, Valencia, CA, USA) and the EZ1 robot (Qiagen) according to the manufacturer’s instructions. Nucleic acid was eluted in a final volume of 60 µL elution buffer.

The purified nucleic acid for each virus was amplified by whole transcriptome amplification (Sigma-Aldrich, St. Louis, MO, USA) according to the manufacturer’s instructions. Amplified cDNA was processed for sequencing on the MiSeq Desktop Sequencer (Illumina, San Diego, CA, USA) using the Apollo 324 System (Wafergen Biosystems, Fremont, CA, USA) and the PrepX ILMN DNA Library Kit (Wafergen Biosystems). After sequencing on the MiSeq, the resulting reads were processed using CLC Genomics Workbench (CLC Bio, Boston, MA, USA). Reads were quality trimmed and aligned against the EBOV reference sequence (Ebola virus/H.sapiens-tc/COD/1976/Yambuku-Mayinga; GenBank number NC_002549.1) or (Ebola virus/H.sapiens-wt/SLE/2014/Makona-G3831; GenBank number KM233103). The trimmed reads were then mapped against this initial consensus sequence to generate a final consensus sequence for each isolate used in this study. The GenBank accession # for the two isolates are: Zaire_1 (Makona) KR824525; Zaire_2 (Kikwit) KR824526. The sequence reads have been submitted to the SRA library under the project ID: PRJNA284630.

### 2.2. Real-Time RT-PCR Assay Evaluations

All EBOV assays, irrespective of the original design format (conventional end point, TaqMan probe or MGB probe, *etc.*), were tested in a SYBR Green assay format under one PCR conditions. The two templates used in the assays were: EBOV Kikwit (GB accession #KR824526) and Makona EBOV (2014 GB accession #KR824525). The mismatches if any in the primer binding sites in each of the templates are listed in Table 2. 

Primers for each assay were purchased from Life Technologies. All real-time RT-PCR assays were performed with primers at a final concentration of 1.0 µM and SYBR Green at 0.2× using the SuperScript One-Step RT-PCR Kit (Life Technologies) at 1×, 3 mM MgSO_4_, and 1× RT/Platinum Taq polymerase. Bovine serum albumin was also added at a final concentration of 0.25 mg/mL. All assays were run on the LightCycler 480 (Roche Applied Science, Indianapolis, ID, USA). Assays that produced an amplicon less than 200 bp were conducted at the following cycling conditions: 50 °C for 15 min (1 cycle); 95 °C for 5 min (1 cycle); 95 °C for 1 s, 50 °C for 20 s and 72 °C for 1 s (45 cycles). A single fluorescence read was taken at the end of each 72 °C step. Those assays with amplicons larger than 200 bp had an extension step of 30 s at 72 °C. 

Preliminary LoDs were determined for each assay using 1:10 serial dilutions of EBOV Kikwit and EBOV Makona RNA. A nonlinear regression analysis was conducted over the linear range of the RNA dilution series for each assay to compare differences in the y-intercept and the slope between the Kikwit and Makona viral RNAs. Ebola Zaire-TM and Ebola Zaire-MGB assays were run according to published protocols [22].

### 2.3. PCR Signature Erosion Tool (PSET) Analysis 

Primers, probes, and amplicons from all PCR assays (see Table S1 for a list of the assays) were analyzed using bioinformatics tools to identify potential false-positive and false-negative matches to publicly available sequences. BLAST+ was used to compare the assay sequences against four NCBI nucleotide databases (nt, wgs, env_nt, and gss), which encompass all non-redundant sequences including whole genome shotgun sequences and environmental sequences. The BLAST+ output was automatically filtered and processed using a pipeline of tools and scripts. The final results were manually validated, and true and false matches were reported. For this analysis, PSET was run on a single node of a cluster with 24 processors. The processing time was mainly determined by the BLAST+ searches and was a few hours for several assays and more than a day for 20 assays. The BLAST+ parameters were optimized for better alignment with short sequences and to allow for a very large number of returned hits so that all true positives were returned. The “gapextend” parameter was set to 4, the “gapopen” parameter was set to 0 and the “word_size” was set to 8 to aid in aligning to short sequences. The “num_descriptions”, “num_alignments,” and “e-value” parameters were all set to 40,000 to allow for many hits and ensure that no true-positive match to a short sequence would be missed. When searching for sequences that were particularly short (<18 bp) the “e-value” parameter was set to 1.5e^−5^. 

To aid in the interpretation of the results of the primer and probe set analysis, a few key definitions and assumptions are provided here. A positive match between the assay primer set sequences and sequences from the NCBI databases at 90% identity over 90% of the primer length is defined as an “assay hit.” An amplicon sequence from an assay is considered an ‘amplicon hit’ if it matches at 85% identity over 90% of the amplicon length. By comparing the number of assay hits with the number of amplicon hits, the final results can be labeled descriptively. If an assay and its amplicon both have a hit to a target organism, it was marked as a true-positive. If an assay and amplicon both have a hit to an unintended organism, it was marked as a false-positive. If there were fewer assay hits for the intended organism, the amplicon hits were labeled as false-negatives. If there were more amplicon hits to unintended organisms than assays hits, these were labeled as true-negatives.

All false-negative results were manually validated. Occasionally, SNPs or indels (especially in short primers or probes, or at the primer ends) prevented the assay from being reported in the BLAST+ results. Validation included examining the pairwise alignment files for the amplicons and visually inspecting how many SNPs and indels were present in the specific primer or probe sequences. For example, if a SNP is found at the 2^nd^ to last base pair of a primer, the last two base pairs are typically omitted from the reported BLAST+ hit. Since some of the primers and probes are very short this could cause the results to fail the 90% identity over 90% of the length requirement, although in the laboratory the assay would still likely work. Based on this assumption, these results were manually changed to positive hits in the results files. Visualization of percentage differences in individual assay and amplicon hits against each GenBank entry was done by generating heat maps using R [23]. 

### 2.4. Bio*Velocity* Analysis for Identifying Diagnostic Signatures

One method for the rapid generation of new diagnostic assays is to use high performance computing to find highly conserved and unique regions of genomes. Bio*Velocity* is a bioinformatics tool based on an innovative algorithm and approach to genomic reference indices. Using a fast and accurate hashing algorithm, Bio*Velocity* is capable of quickly finding conserved and signature sequences from within a given genomic reference or set of raw sequencing reads. This process allows one to find target regions for new assay design within a particular genus, species, or strain in minutes (to be published elsewhere; Bio*Velocity* will also be available in the Illumina App Store in June 2015).

Bio*Velocity* takes advantage of a CRAY-XMT2 supercomputer with four terabytes of RAM to demonstrate dramatic improvements in performance over current technologies with 50× faster speeds, increased functionality, increased throughput, and improved accuracy. The CRAY-XMT2 allows for the use of a brute force index, built out of all possible base pair sequences of various k-mer lengths. This index is used to map against thousands of references and allows for quick alignment of k-mers amongst them simultaneously. 

Once a target is selected, in this case EBOV, the conserved regions must be isolated. To determine conserved regions Bio*Velocity* needs two sources of data: a list of full genome references of the desired target organism and a k-merized genome reference or read set of the same target organism. Using Bio*Velocity*, an index was created using all available EBOV whole genome references, 134 in total from NCBI (as of December 2014). Conserved sequence sizes can be manually set based on the necessary application. For example, to find conserved sequences that are 50 base pairs in length, a single full genome reference is split into 50-mers and Conserved Sequence Domain (CSD) detection pipeline is run against the index. Bio*Velocity* scans all 50-mer alignments to identify perfect match in every reference sequence located within the index. If a particular 50-mer perfectly aligned to each of the 134 EBOV references it would be considered conserved and exported into a FASTA file of conserved k-mers. 

Signature Sequence Domain (SSD) detection pipeline is designed to compare one organism, either a reference genome or read set, to all other references found within the index to determine unique k-mers. Similar to CSD, SSD uses Bio*Velocity*’s index to quickly align k-mers against thousands of references, but as opposed to CSD, SSD looks for k-mers which have no perfect alignments against any reference genome within the index. After running CSD, Bio*Velocity* collects all conserved k-mers for an organism (e.g., EBOV) and uses this newly acquired k-mer set to determine signature k-mers through the alignment against an index comprised of all of NCBI’s full genome references (excluding the target organism, EBOV). Thus, a set of signature k-mers for the target of interest, EBOV, is generated. These k-mers are prime locations for assays to target due to their conserved status, while also minimizing likelihood of false-positives since they are unique to the target.

The detection pipelines for both CSD and SSD were run for EBOV to determine how many potential signature k-mers are available to target new assay design. The results are shown both against all viruses in NCBI and against all organisms in NCBI (Table S3). The first column indicates the k-mer size used, the second column indicates the conserved sequences found in EBOV, and the following columns indicate the number of conserved sequences that are also signature sequences based on varying degrees of similarity. For instance, a signature found at 90% means that there are no other sequences that are similar at a 90% or greater level in the index. This is useful for ensuring that target regions can sustain some genomic plasticity before losing their signature status.

### 2.5. Validation of Bio*Velocity* Signatures

BLAST (NCBI) was used to identify ebolavirus orthologs to each gene (NP, VP35, VP40, GP, VP30, VP24, and L), using EBOV AF086833 as the initial comparator sequence. The BLAST program blastn was used with parameters limiting the search to taxid ebolavirus, and the maximum number of sequences returned set to 250. The e-value maximum was set to 100, and the word size was 11. Because of the diversity among ebolaviruses, the match/mismatch penalties were set to 1, −1, and the gap penalties were set to 0 for existence and 2 for extension. The BLAST results were saved in FASTA format and aligned using ClustalW in BioEdit with some manual editing of the alignments. The precise signature regions were selected after alignment and the ranges of percent identity per species were calculated using BioEdit.

## 3. Results 

### 3.1. Performance Assessment of Currently Available EBOV PCR Assays Using PCR Signature Erosion Tool (PSET) 

Gire *et al.* reported that some of the EBOV diagnostic assays that were developed prior to the 2014 outbreak may not perform efficiently in detecting the 2014 EBOV isolates due to sequence variations in the PCR targets in the new viral sequences [15]. This analysis did not take into consideration the PCR chemistries or the possibility that the assays may perform even with the changes in the target sequences, which may or may not impact detection depending on the position of mismatches between the primers and the targets [24]. As a first step towards addressing this issue, we systematically evaluated signature erosion *in silico* and its potential effect on assay performance. The various EBOV assay signatures were compared against all the ebolavirus sequences available in GenBank (this included complete whole genome sequences as well gene sequences) in order to determine the variability in the target sequences and the potential effectiveness or failure of these assays. In this *in silico* approach (termed PSET), we not only searched for perfect matches to the assays’ signatures but also wanted to capture additional potential false-positive and false-negative matches by relaxing the identity to 90% along 90% of the length of primer/probe sequences. In order to minimize the number of false-negative results, we relaxed the identity criteria for those amplicons to 85% along 90% of the length of the amplicon. In this approach, we can descriptively define the true-positive, true-negative, false-positive, and false-negative matches. For example, if there is an assay hit (indicated by matching primers/probe) and an amplicon hit (indicated by the presence of an amplicon of the expected size) in the intended organism’s sequence, it is marked as a true-positive. If there is an assay hit, (indicated by matching primers/probe) and an amplicon hit (indicated by the presence of an amplicon of the expected size), in an unintended organism’s sequence, it is marked as a false-positive. If there is no assay hit but an amplicon hit for the intended organism’s sequence, it is labeled as false-negative. If there is no assay hit for the unintended organism whether or not there is an amplicon hit, it would be labeled as true-negatives.

**Figure 1:**
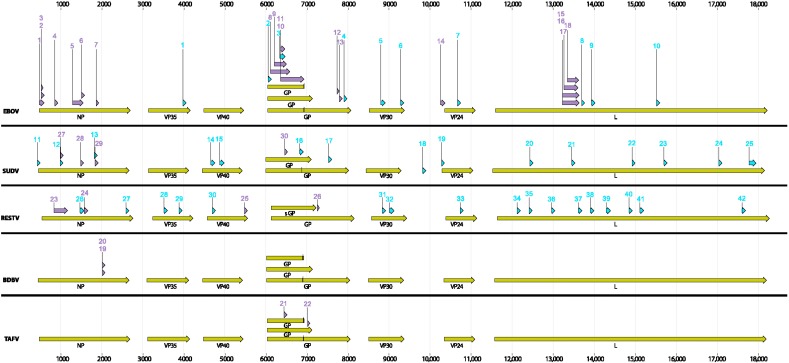
Genome maps of five members of the *Ebolavirus* genus (drawn to scale). The various genes (green arrows) and the location of the PCR amplicons of the current assays (purple arrows), and the Bio*Velocity* generated signatures (blue arrows) are marked. The numbers of the purple arrows correspond to the assay numbers described in Table S1 and those of the blue arrows correspond to sequences described in Table S4.

For PSET analysis, we took 16 published and two unpublished EBOV-specific RT-PCR assays and queried four different nucleotide databases in NCBI for possible matches. Among the 18, six assays that had ambiguous bases in the primer sequences (degenerate primers) were not considered for this analysis since the current version of the PSET algorithm cannot handle such degenerate sequence positions. We also included 12 non-EBOV assays that target the other four species of the *Ebolavirus* genus. The assays and pertinent sequence information along with the references where available are presented in Table S1. The location of the various PCR targets on the respective genomes is indicated in Figure 1. For this analysis, PSET was run on a single node of a cluster with 24 processors. The processing time was mainly determined by the BLAST+ searches and was a few hours for several assays and more than 24 h for 20 assays. 

The assay hits and amplicon hits for each assay against the five members of the *Ebolavirus* genus are summarized in Table 1. Some of the highlights of this analysis include: (1) Most of the EBOV-specific assays (10/12) identified the EBOV sequences as expected. These are marked as the number of hits in the true-positive column. One of the assays that failed ((Ebo-GP-1 (#10)), produced 12 false-positive hits to RESTV and the second EBOV specific assay that failed [Ebola Zaire-TM (#13)] produced nine false-negative results; (2) Among the 12 non-EBOV assays 11 matched their expected targets and the one assay that failed (Ebola Reston-MGB (#26)) produced one false-negative result. These results are based on the 90/90 and 85/90 rule for assay hit and amplicon hit respectively; (3) There are also the cases where the assays failed the primer/probe match criteria but passed the amplicon match criteria and, thus, were marked as true-negatives. These included off-target matches of the ampliocns of EboZNP (#4), Ebola MGB-RESTV (#24) against EBOV, Ebola Sudan-MGB (#27) against EBOV, and RESTV assays; (4) The last category, produced assay matches but no amplicon hits at the set thresholds (EBO-GP-1 (#10) against RESTV, FiloAB (#16) against MARV and SUDV sequences)). This is due to the fact that variations in these sequences are more than the 15% cutoff set by the criteria for amplicon hits. These amplicon sequences were extracted from GenBank and verified that indeed the variation was more than 15% ((EBO_GP-1 has 211 SNPs over 580 bases (37%) and FiloAB has 136 SNPs over 418 bases (33%)). Since the number of assays that yield this result is very small, and these results can be confirmed manually, we have chosen to keep the PSET threshold for amplicons at 85% to minimize the number of false-negatives that would have to be validated. In this case, these assays are meant to be pan-assays and were labeled as true-positives. 

The true-positive hits that passed the criteria are based on 90/90 and 85/90 rule for assay hit and amplicon hit, respectively. We calculated the number of perfect assay hits (100/100) and amplicon hits (100/100) for each assay. They varied from 0% to 100% depending on the assay and number of target sequences (Table 1); only three out of 24 assays passed these criteria. Thus, setting a lower identity threshold for PSET allows for capturing a broader set of sequences that may produce positive assay results since, depending on the location of the mismatches in the primer or probe sequence and the PCR assay conditions, there may not be any effect on the assay performance (see below empirical data for EBOV assays).

**Table 1:** PCR Signature Erosion Tool (PSET) analysis of ebolavirus PCR assay signatures.

Assay #	Assay ID	Intended Target Species	Gene Target	Species/Strain of Hits	Amplicon Length (bps)	Assay Hit	Perfect Assay Hits	Percentage of Perfect	Amplicon Hits	Perefect Amplicon Hit	Percentage of Perfect	True-Positive	False-Negative	False-Positive	True-Negative	Report *
1	Sig 1	EBOV	NP	EBOV	124	135	132	97.8	135	11	8.1	135	0	0	0	Pass
2	EbolaZaire-MGB	EBOV	NP	EBOV	76	135	134	99.3	135	124	91.9	135	0	0	0	Pass
3	Sig 3	EBOV	NP	EBOV	49	135	135	100.0	135	125	92.6	135	0	0	0	Pass
4	EboZNP	EBOV	NP	EBOV	80	136	22	16.2	136	19	14.0	136	0	0	0	Pass
4	EboZNP	EBOV	NP	SUDV	80	0	0	0	11	0	0	0	0	0	11	
5	ZAI-NP	EBOV	NP	EBOV	268	148	123	83.1	148	10	6.8	148	0	0	0	Pass
6	Ebola MGB-EBOV	EBOV	NP	EBOV	79	148	23	15.5	148	11	7.4	148	0	0	0	Pass
7	ENZ	EBOV	NP	EBOV	70	148	32	21.6	148	32	21.6	148	0	0	0	Pass
10	EBO-GP-1	EBOV	GP	EBOV	579	152	0	0	152	5	3.3	152	0	0	0	Fail
10	EBO-GP-1	EBOV	GP	RESTV	579	12	0	0	0	0	0	0	0	12	0	
12	ZebovGP	EBOV	GP	EBOV	64	153	13	8.5	153	13	8.5	153	0	0	0	Pass
13	Ebola Zaire-TM	EBOV	GP	EBOV	80	144	13	9.0	153	13	8.5	144	9	0	0	Fail
16	Filo AB	pan-Filo	L	EBOV	419	135	0	0	135	12	8.9	135	0	0	0	Pass
16	Filo AB	pan-Filo	L	MARV	419	55	0	0	0	0	0	55	0	0	0	
16	Filo AB	pan-Filo	L	SUDV	419	12	0	0	0	0	0	12	0	0	0	
17	GAB-1	EBOV	L	EBOV	353	135	17	12.6	135	12	8.9	135	0	0	0	Pass
19	Ebola BDBV-MGB	BDBV	NP	BDBV	74	5	1	20.0	5	1	20.0	5	0	0	0	Pass
20	Ebola BDBV-TM	BDBV	NP	BDBV	74	5	1	20.0	5	1	20.0	5	0	0	0	Pass
21	Ebola TAFV-MGB	TAFV	GP	TAFV	64	2	1	50.0	2	1	50.0	2	0	0	0	Pass
22	Ebola TAFV-TM	TAFV	GP	TAFV	79	2	2	100.0	2	2	100.0	2	0	0	0	Pass
23	Reston	RESTV	NP	RESTV	337	8	8	100.0	8	3	37.5	8	0	0	0	Pass
24	Ebola-MGB-RESTV	RESTV	GP	RESTV	97	8	2	25.0	8	2	25.0	8	0	0	0	Pass
24	Ebola-MGB-RESTV	RESTV	GP	EBOV	97	0	0	0	9	0	0	0	0	0	9	
25	Ebola Reston-TM	RESTV	VP40	RESTV	80	8	2	25.0	8	2	25.0	8	0	0	0	Pass
26	Ebola Reston-MGB	RESTV	GP	RESTV	55	11	10	90.9	12	10	83.3	11	1	0	0	Fail
27	Ebola Sudan-MGB	SUDV	NP	SUDV	80	11	10	90.9	11	10	90.9	11	0	0	0	Pass
27	Ebola Sudan-MGB	SUDV	NP	RESTV	80	0	0	0	8	0	0	0	0	0	8	
27	Ebola Sudan-MGB	SUDV	NP	EBOV	80	0	0	0	148	0	0	0	0	0	148	
28	Ebola MGB-SUDV	SUDV	NP	SUDV	81	11	7	63.6	11	2	18.2	11	0	0	0	Pass
29	Sudan	SUDV	NP	SUDV	89	11	10	90.9	11	10	90.9	11	0	0	0	Pass
30	Ebola Sudan-TM	SUDV	GP	SUDV	77	14	9	64.3	14	9	64.3	14	0	0	0	Pass

* Pass/Fail call for each assay based on 90/90 and 85/90 rule for assay hit and amplicon hit, respectively. Number of sequences is limited in non-Zaire assays and, hence, does not represent the true genetic diversity of the species and, hence, the true success of the non-Zaire assays.

**Figure 2:**
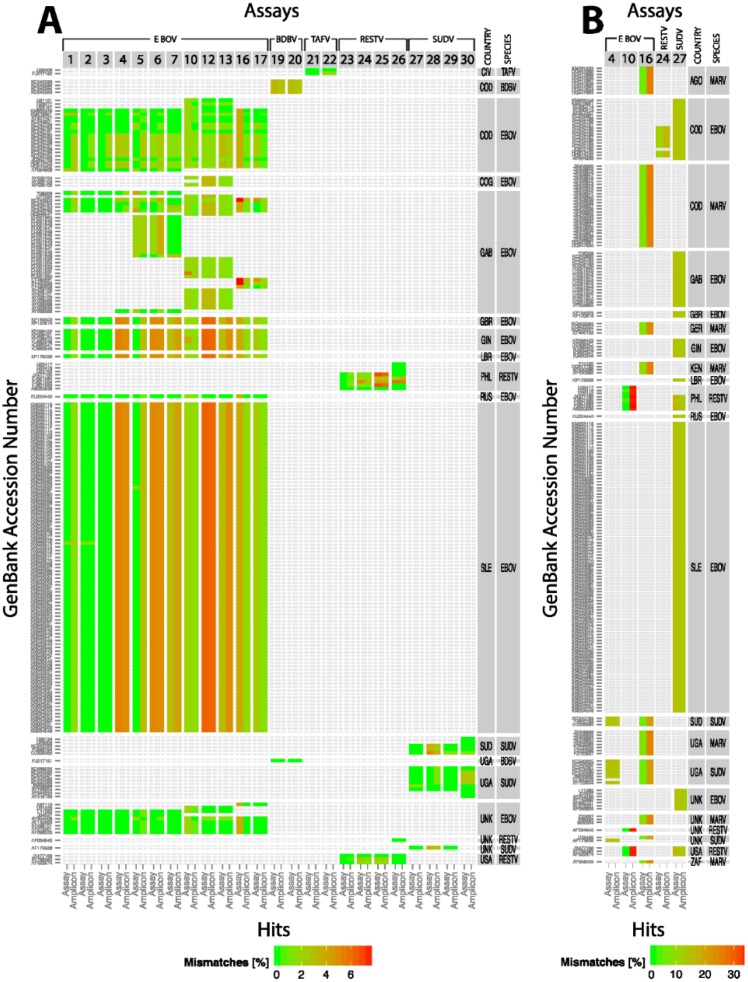
Heat map of assay and amplicon hits for each assay based on percentage mismatches between the reference and various GenBank sequences. The various assays, numbered in the same order as in Table S1, are grouped according to specificity of the assay; *i.e.*, EBOV (1–17), BDBV (19–20), TAFV (21–22), RESTV (23–26), and SUDV (27–30). The GenBank accession numbers are provided on the left, and the corresponding ebolaviruses and isolation countries are labeled on the right. The country of isolation is based on the information provided in GenBank entries. Country codes: AGO-Angola; CIV-Ivory Coast; COD-Democratic Republic of Congo; COG-Congo; GAB-Gabon; GBR-United Kingdom; GIN-Guinea; LBR-Liberia; PHL-Philippines; RUS-Russian Federation; SLE-Sierra Leone; SUD-Sudan; UGA-Uganda; UNK-Unknown; USA-United States of America; ZAR-South Africa. Assays/amplicon hits that match at 100/100 rule are in green, and others with various percent mismatches are color coded according to the scale at the bottom. (**A**) represents the sensitivity (color) and specificity (species) of true-positive hits to the respective assays; (**B**) represents the sensitivity (color) and lack of specificity (cross reactivity) to the respective assays. False-positive (#10, #16), true-negatives (#4, #24, #27). Note that the heat index scale is different in panels A and B.

In order to more quantitatively define the sensitivity and specificity of these assays against every GenBank match, a heat map was generated [23]. In this case, sensitivity for each assay is represented in the heat map as the percentage of mismatches in individual GenBank sequences against signatures (primers/probe/amplicon) (Figure 2a representing true-positives), and the lack of *specificity* of the assays is represented by the reactivity of non-target organisms for any given species-specific assay (Figure 2b). The primary data used to generate the heat map is presented in Supplementary Table S2. The *sensitivity* level is indicated by the color; *i.e.*, the greenest features represent perfect assays with 0 mismatches with the shift towards red being not optimal or poor assays containing mismatches (Figure 2a,b) which varied with different assays. In general, species-specific assays reacted to the appropriate species sequences with a few exceptions (see below). The assays that cross-react to other species are presented in Figure 2b. The assays that did not produce an assay hit but an amplicon hit (true-negatives) (EboZNP (#4), Ebola MGB-RESTV (#24) and Ebola Sudan-MGB (#27) and those assays that produced an assay hit but did not produce an amplicon hit because of a failure to pass the 85/90 threshold are represented in panel B of the heat map; (EBO-GP (#10) against RESTV and FiloAB (#16) against MARV and SUDV) (Figure 2b). In the latter case, EBO-GP produced false-positive results while FiloAB hits were marked as true-positives since this pan assay is intended to detect all filoviruses.

### 3.2. In Vitro Assessment of the Performance of EBOV RT PCR Assays Using Kikwit and Makona EBOV RNA 

In addition to the *in silico* analysis of the assay signatures, we analyzed the currently available EBOV RT-PCR assays. For this analysis, we took 16 published assays and compared their performance against two templates [EBOV Kikwit (1995) and Makona (2014) under standardized rRT-PCR conditions (SYBR green assay). Both viruses were sequenced on the MiSeq Desktop Sequencer. The average depth of coverages for the EBOV Makona isolate was 219× (27,971 reads mapped) and for EBOV Kikwit isolate was 690× (88,824 reads mapped). Both viruses had at least 5× breadth of coverage over the entire length of the genome, excluding the 5′ and 3′ ends. The EBOV Kikwit was nearly identical to the EBOV Kikwit sequence in GenBank (AY354458), having a single nucleotide change at position 7327 (C7327T). For the Makona isolate, no new mutations were identified (compared to the 300 Makona whole genome sequences within GenBank as of 18 May 2014) with the exception of a non-synonymous change (C7660T; note this variant was in a low coverage region (36×), and 15% of the paired reads had the C at position 7660).

**Table 2:** SYBR GREEN qRT-PCR results of EBOV Assays (all except the last two are SYBR green assays).

Assay #	Assay Name	Gene	Forward Primer	Reverse Primer	Amplicon	Kikwit SNPs		Kikwit Assay Metrics		Makona SNPs		Makona Assay Metrics	
			nts	nts	bps	Primer 1	Primer 2	Cq	LoD	Primer 1	Primer 2	Cq	LoD
1	Sig 1	NP	21	22	124	None	None	31.17 ± 0.51	100	None	None	29.96 ± 0.47	100
2	Ebola Zaire-MGB	NP	24	23	76	None	None	27.11 ± 0.09	100	None	None	26.77 ± 0.08	100
3	Sig 3	NP	24	17	49	None	None	26.98 ± 0.08	100	None	None	26.63 ± 0.06	100
4	EboZNP	NP	27	21	80	None	None	26.82 ± 0.10	100	A4G,A6G	A8G	26.91 ± 0.07	100
5	ZAI-NP	NP	26	22	268	None	A3G	28.91 ± 0.11	100	None	None	28.32 ± 0.18	100
6	Ebola MGB-EBOV	NP	20	23	80	None	None	23.72 ± 0.10	1000	A1G, A13T	C23A	24.66 ± 0.02	1000
7	ENZ	NP	18	20	70	None	None	23.43 ± 0.01	1000	A9G	None	24.07 ± 0.09	1000
8	EBO1/2	GP	20	20	479	None *	None *	29.50 ± 0.49	1000	None *	None *	25.74 ± 0.65	10,000
11	EBO-GP-2	GP	21	20	112	None *	None *	27.88 ± 0.07	100	None *	None *	27.50 ± 0.07	100
12	ZebovGP	GP	19	22	64	None	A16G	24.59 ± 0.05	1000	G10A	G7T, T10C	25.17 ± 0.12	1000
13	Ebola Zaire-TM	GP	23	20	80	None	G14A	27.29 ± 0.03	100	None	G14A	27.5 ± 0.05	100
14	KGH	VP24	20	20	122	None	R15A	25.50 ± 0.03	100	None	R15G	25.02 ± 0.05	100
16	Filo AB	L	22	30	419	T8C	G10A	30.96 ± 0.17	100	None	T4C, G10A	30.34 ± 0.14	100
17	GAB-1	L	21	21	353	None	None	26.84 ± 0.07	100	C16T	None	26.87 ± 0.11	100
13^+^	Ebola Zaire-TM	GP	23	20	80	None	G14A	34.12 ± 0.14	10	None	G14A	34.05 ± 0.42	10
2^++^	Ebola Zaire-MGB	NP	24	23	76	None	None	33.57 ± 0.18	10	None	None	36.91 ± 0.54	1
^+^ TaqMan assay- probe mismatch in Makona G9A													
^++^ MGB probe assay- probe is fine in both templates													

LoD (putative): (pfu/mL); * degenerate primers

**Figure 3:**
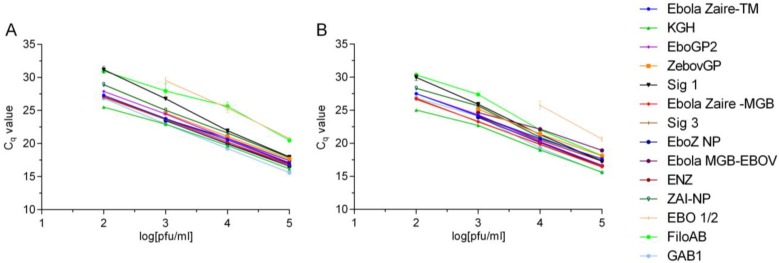
SYBR Green RT-PCR assay results as a function of pfu/mL *vs.* Cq (cycle of quantitation). Each individual assay was run with a 1:10 serial dilution of either EBOV Kikwit (**A**) or EBOV Makona (**B**). Each PCR was run in triplicates, and the lowest virus concentration indicated is the lowest virus dilution that still resulted in all three replicates being called positive. For each assay, a sample was considered positive if the Cq value was less than the average of two no template controls. The error bars indicate the standard deviation of the mean.

The results of these analyses are summarized in Table 2 and Figure 3. The results indicated that there are differences in assay performance between different assays as indicated by the variations in Cq and LoD (compare for example the Ebola Zaire-MGB *vs.* EBO1/2); however, with some exceptions, there were minimal differences in assay performance between the templates, despite the fact that there are some mismatches in the primer binding sites in some assay signatures. Some assays exhibited the same efficiency with mismatches in the primers (assay #4, 5, 7, 17, and 13 in TM format) and different mismatches with the same efficiency (assays #12 and 16). In all these cases, the position of the mismatches are such that they had a minimal effect as shown in other studies [24]. Similarly, assay #6, despite a mismatch at the 3’ terminal position (C23A), had a similar efficiency with both templates. Others previously demonstrated that certain mismatches at the 3′ position in the reverse primer had minimal, if any, effects on quantitative RT-PCR amplification with mismatches at the 3′ end of the forward primer can have a critical impact [24]. There is one assay each with the same mismatch (#8) or no mismatch (#2 in MGB format) but different efficiencies. For assays #6 (EBO1/2) and #2 (Ebola Zaire-MGB in MGB format), the differences in the preliminary LoDs are likely not significant. The differences in the Cq values between the LoDs are approximately 3.3, the expected difference for a 1:10 dilution series. It is likely that, when conducting a confirmation of LoD with 60 replicates at the LoD, several replicates would not be positive at the lower dilution, and hence the true LoD would be potentially 10x higher. Although the different assays were not tested here under the formats and conditions they were originally designed for and intended to be tested, the overall performance of the assays indicate that the mismatches do not impact the assay performance to any significant extent. In addition two of our published assays (Ebola Zaire-TM and Ebola Zaire -MGB assays) were tested in their respective configuration with TaqMan and MGB probes using Kikwit and Makona templates. For the Ebola Zaire-TM assay, the LoD was 10 pfu/mL for both Kikwit and Makona. The LoD for the Ebola Zaire-MGB assay was 1 pfu/mL with Makona and 10 pfu/mL with Kikwit templates. The difference in these LoDs is within the experimental variation of the assay and would likely be the same when running a confirmation of LoD using more replicates (60 replicates). The published LoDs for the Ebola Zaire-TM and Ebola Zaire-MGB assays were 0.02 pfu/mL and 0.2 pfu/mL respectively [22]. This difference could be attributed to the differences between viral preparations and the differences in genomic equivalence to pfu [25]. 

The significant finding is that the Ebola Zaire- MGB assay, despite having a perfect match between the assay signatures and the 2014 EBOV target sequences, was reported to be 100× less sensitive in an end point PCR assay in clinical matrix compared to KGH assay [15]; however, in the real time PCR format tested in buffer in this study, we did not find any difference between KGH and Ebola Zaire-MGB using Kikwit or Makona template.

### 3.3. Discovery of Novel Assay Signatures Using a Whole Genome Approach and a Novel Algorithm Termed BioVelocity

Given the diversity seen in EBOV sequences and the recent expansion in the number of EBOV whole genome sequences, we wished to identify new assay signatures based on all available whole genome sequences of EBOV. We used Bio*Velocity,* a platform that provides very rapid comparisons among large numbers of sequences utilizing a novel hashing algorithm, termed Bio*Velocity* [26]. Bio*Velocity* first finds regions that are conserved in the target organism (CSD-conserved sequence domain) from a library of all whole genome sequences of the target organism and then compares the identified conserved regions to all other viruses and all organisms in GenBank, to exclude non-unique conserved regions. The run-time for this analysis is about 20 min. The remainder of the conserved regions are considered signatures for the target organism (SSD-signature sequence domain). The CDD analysis started at a 20-mer length at 100% identity and incrementally increased to the greatest conserved length, which was 146-mer for EBOV. To remove non-unique conserved regions during the comparison to the all-viral and all-GenBank sequences, we first set criteria at 100% match to other organisms, and then incrementally decreased the match criteria to 80% to assess the effect that variations in the reference genomes would have on the number of signatures, thus applying a stricter standard for target organism signatures. 

The initial step of this analysis identified conserved regions in the target organism through an analysis of a library of the target organism genomes; thus, the number of conserved regions identified is inversely proportional to the population diversity represented by the reference library of the target organism. The better the diversity is represented, the more robust are the signatures. 

**Figure 4:**
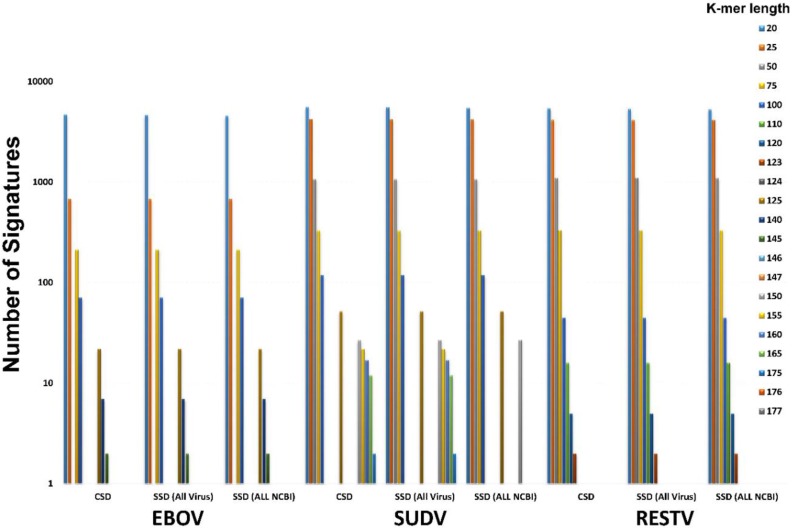
Various ebolavirus-specific Conserved Sequence Domains (CSD) *vs.* Signature Sequence Domains (SSD) identified using Bio*Velocity*. The bars are color coded according to the k-mer size.

**Figure 5:**
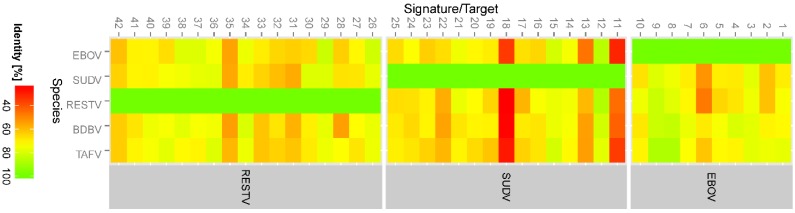
Heat map of percentage identity of Bio*Velocity* signature hits in various ebolaviruses. The numbers on the *x*-axis represent species-specific signature IDs (EBOV: 1–10; SUDV: 11–25; RESTV: 26–41). The number of hits for each species for any given signature is given in Table S5. As expected the species-specific signatures are highly conserved in their respective species (indicated by green) compared to other species. The heat index on the left shows the percentage identity.

Using this approach we discovered ten unique signatures, large enough to be used to develop PCR assays that are present and unique to EBOV at 100% conservation. The results of this analysis, *i.e.*, number of features as a function of k-mer size are presented in Figure 4. The sequences of the best candidate Bio*Velocity* (BV) signatures and their lengths are presented in Table S4 and their respective locations on the reference genome in Figure 1. In order to validate that these signatures are unique to EBOV, we used BLAST analysis to query these regions in all *Ebolavirus* sequences (Table S5) and a summary of the results is presented in the form of a heat map (Figure 5). None of the ten signatures are present at 100% identity in any of the other species. The identities varied from 46%–88% depending on the signature, target genes, and species, indicating that these are potentially useful EBOV specific PCR signatures. Interestingly, none of the current PCR assay targets tested by PSET overlapped with the Bio*Velocity* signatures except two (EBOV_9 and EBOV_10) which partially overlapped the BV signatures in this region (Table S5, Figure 1). 

We extended this approach to find signatures specific for other ebolaviruses. The results are presented in Table S4 and Figure 4. As with EBOV, we identified 15 and 17 species-specific signatures in RESTV and SUDV, respectively. TAFV and BDBV did not have adequate number of reference whole genome sequences, and therefore yielded non-productive, (*i.e.*, non-robust) conserved regions. Essentially, there were no conserved regions since the entire genome was returned as a conserved domain. It is apparent from this analysis that the number of conserved features among the sequences of the index organism is primarily determined by the library size; *i.e.*, with more sequences, the number of CSDs decrease, and these CSDs tend to be SSD when probed against other viral sequences or all organisms (Figure 4). Thus, it is critical to expand the index library of sequences for any given species in order for Bio*Velocity* to be an effective tool for identification of diagnostic signatures. It is worthwhile to note that we could not find any pan-specific CSD or SSD due to extensive genetic diversity among the five ebolaviruses.

## 4. Discussion 

Rapid and accurate diagnosis of the etiological agent present in a clinical sample is critical for appropriate responses including patient triage and outbreak control measures. Accurate detection also curtails disease transmission by preventing false-positive and false-negative misdiagnoses that may result in catastrophic consequences. Highly sensitive and specific assays also help to monitor response to therapy by quantitation of viral load during treatment and to assess the efficacy of therapeutics currently undergoing clinical trials. The current gold standard for EBOV diagnosis is rRT-PCR, and accurate diagnosis of the presence of EBOV in clinical samples relies on optimal RT-PCR assay kinetics.

Whole genome sequence analysis of the 2014 EBOV viral samples unraveled a major vulnerability of some of the current diagnostic assays due to genetic changes in the RT-PCR target genes [15]. We therefore sought to perform quantitative comparisons of various existing assays before drawing any conclusion about potential inefficiencies of the current assays or design and performance of new assays. In the current study we took an empirical approach to evaluate many of the RT-PCR assays using the 2014 EBOV viral genomic material as a template in comparison to EBOV Kikwit. This analysis indicated varying efficiencies and sensitivities between the assays using the same RNA template and assay conditions. In fact, two of the currently fielded assays developed prior to the onset of the 2014 outbreak (one has less than 100% signature identity to the 2014 EBOV sequences) performed equally well compared to EBOV Kikwit template. These assays are being successfully used in clinical diagnostics in Western Africa. Most notably, we found minimal, if any, difference in assay performance between the old and new EBOV templates, giving confidence in the results of the diagnostic tests.

We also took an *in silico* approach to assess the effectiveness of the assays against all the available filovirus sequences in GenBank. This analysis indicated that out of the 24 assays analyzed only three assays showed perfect matches to their target sequences. All the other assays showed potential false-negative results. If the identities of the assay sequences were relaxed to 90/90, many of the assays met the standard. In other words, these assays would be functional depending on the location of the mismatches. Three assays failed even at this relaxed criteria due to false-positive or false-negative results. False-positive results, *i.e.*, an assay meant to detect EBOV that also detects other members of ebolavirus may be acceptable in the case of EVD since the treatment options are the same for EVD caused by any ebolavirus. But there are other instances when false-positive results would result in unnecessary treatments, e.g., misdiagnosis of EVD in the setting of another viral infection with different transmission dynamics, treatment, and prognosis. However, false-negative results, *i.e.*, misdiagnosis or non-diagnosis can have serious negative consequences. 

Given the rapid accumulation of mutations in ebolavirus genomes and other RNA viruses, systematic and periodic *in silico* evaluation of the current assay signatures against newly added sequences in public databases is crucial for deploying effective diagnostic tests for reemerging pathogens [18]. Empirical testing of assays may not be feasible for every new isolate due to difficulties in obtaining and testing materials under the requisite containment levels in a rapid time frame. 

It is well established that the introduction of EBOV in the current and past human outbreaks was from zoonotic reservoirs [9,10]. Hence, real-time biosurveillance of zoonotic sources in known hot spots of EVD may aid in preventing an outbreak in humans before it occurs. Biosurveillance may also allow tracking the changing genetic profiles of the virus itself in the environmental reservoir. Biosurveillance can be accomplished using currently available molecular assays and the genomic changes can be tracked by using unbiased high throughput sequencing of environmental samples using next-generation sequencing. 

In this context, capturing the genetic diversity of geographically and temporally diverse hemorrhagic fever viruses is paramount for assessing and maintaining the effectiveness of assays deployed. As shown in the Bio*Velocity* analysis performed in this study, lack of sequences representing the genomic diversity may hinder the effective design of diagnostic assays and hence would miss a new genetic variant that emerges as the causal organism of an outbreak. It is imperative that samples from outbreaks are made available in a timely fashion for sequencing [27] and genomic sequence data generated are shared. Immediate release and availability of genomic sequences in public databases would allow for crowd sourcing of data analysis and rapid development of new assays or improvement of old assays. It is worth noting that crowd sourcing played a key part in rapid whole genome sequencing and post sequencing analysis of the outbreak pathogen, as well as in the development of new diagnostics during the 2011 *E. coli* O104:H4 outbreak that originated in Germany [28]. Deep sequencing to decipher quasi-species would also elucidate the genetic trends in population within hosts. Using these data, one can predict the potential impact of genetic drifts on signature erosion and consequent failure of existing assays and develop new signatures if the need arises.

Previous studies documented the impact mismatches can have on real-time PCR efficiency [24,29]. Based on these observations and data presented here, we suggest that an *in silico* analysis be performed on any new genomic sequence available to assess the effectiveness of the current assays. If the assays fails to meet specified quality metrics, a new assay design is definitely warranted. Contingent on the regulatory status of the existing assay, the timeliness and ability to impact the emerging outbreak may have to be considered. Any redesign to the assay will require, at minimum, additional analytical testing, and a regulatory submission to the FDA for review and approval.

Rational design of new assays targeting highly conserved regions of the genomes containing discriminatory sequences is essential for the effectiveness of diagnostic assays. The majority of the existing species-specific assays were designed based on limited sequence information and were gene-specific due to historical or laboratory specific interests. A rational approach would be to use all and ideally a large set of whole genome sequences to identify conserved segments. One such approach is described here using Bio*Velocity* with subsequent *in silico* validation of the identified signatures. To provide not only unique but also robust signatures, the index organism genome diversity needs to be properly characterized. Thanks to the recent explosion in whole genome sequencing, this was possible in EBOV and to lesser extent in SUDV and RESTV due to limited number of sequences available. Identifying similar signatures in other species also requires more genome sequences from temporally and geographically diverse isolates that represent the entire genetic diversity of the species.

There is also the desire to use orthogonal approaches for increasing the confidence of the test results. A number of laboratory developed immuno-assays that complement molecular assays are in use in order to increase the confidence of the diagnostic result. Additionally, multiplexing molecular assays for simultaneous analysis of multiple samples and targets is highly desired in any outbreak scenario. This approach not only addresses issues with genomic change but also diagnosis of pathogens that present with similar symptoms. Enabling high throughput analysis in an outbreak scenario will immensely aid in rapid processing of hundreds of samples. We also would like to draw the attention of the research community that some of the sequence-based next generation sequencing platforms with high-throughput, multiplexing and rapid time to result capability will undoubtedly change the future of diagnostics in an outbreak scenario. A recent study based on the Illumina platform addressed such a possibility using a rapid sequencing approach [30].

Improving current assays to address genomic changes of an emerging threat is of paramount concern demonstrated here and in other studies [15,17]; however, the implications of obtaining regulatory approvals and the timely availability of diagnostics during an active outbreak should also be considered. The Emergency Use Authorization (EUA) authority granted by Congress allows the FDA Commissioner to provide medical interventions (medical devices, diagnostics, therapeutics and vaccines) to the American people and Armed forces, to be used when there are no adequate, approved, and available alternatives. While significantly less onerous than the 510 k process, this regulatory path still requires a rigorous level of testing with respect to sensitivity and specificity for the assays as well as documented manufacturing processes to ensure quality of the product. Amending EUA approved diagnostic test applications that address genomic changes requires additional analytical testing, and review of the testing data by the FDA before a new approval is issued. In this context, a recent report by Kugelman *et al.* raises the issue on the effectiveness of therapeutics and vaccines due to genetic drift [17]. While that report focused on therapeutics, similar inferences can be drawn to the diagnostics in use from the current study. 

In the current EBOV outbreak, the World Health Organization (WHO) expressed a desire to put in place an emergency quality assessment mechanism. According to the WHO statement [11], this is a rapid review process for assessing a diagnostic’s quality, safety, and performance similar to the EUA process. Information sought included the recommended specimen types; evidence of test performance, including sensitivity and specificity; suppliers of critical components or raw materials and services, and data on current inventory and manufacturing capacity and quality. The overarching objective is to guide bulk procurement decisions by WHO and other partners that will get the best available tests to Western Africa in a short time frame [11]. 

The PSET and Bio*Velocity* pipelines described here can be easily adapted to assess signature erosion and assay performance of any virus or any other organism rapidly and develop new assay signatures and improve molecular assays. Currently, efforts are underway to improve molecular assays of various viruses such as Lassa and Crimean-Congo hemorrhagic fever (CCHF) virus and bacteria such as *Burkholderia mallei* and *B. pseudomallei* for which current assays are not optimal with respect to specificity and sensitivity. 

It is quite apparent that developing diagnostic assays for RNA viruses is an arms race: rapid genetic changes (especially in the assay target sequences) in viral genomes necessitate changes in the assays in order to detect that genetic variant. These new signatures in turn decay over time forcing new assay designs. It is hoped that some of the considerations outlined in this study will guide future diagnostics development and raise awareness and initiate a discussion on these and related regulatory issues in light of the current EVD crisis in order to be better prepared for a future emerging or reemerging biothreat. 

## 5. Conclusions

In this study we assessed the performance of existing EBOV assays using *in vitro* and *in silico* (PCR Signature Erosion Tool) approaches. Because viral evolution is dynamic, the genetic diversity of an outbreak species can expand during the course of an epidemic. Just as viral evolution is adaptive, the development of diagnostic tests must be open-ended to identify newly emerging quasi-species, and to mitigate the risks of lost sensitivity and false-negative results. This is particularly relevant to sequence-based diagnostic tests. In addition, flexibility in the regulatory process is essential. An accelerated FDA approval process should exist to account for inevitable changes to molecular assay signatures. In order to increase the confidence of diagnostic assays, orthogonal point of care diagnostics need to be readily available, with a minimal time to develop and deploy.

The rational deployment and development of sequence-based diagnostic tests depends upon WGS done early, quickly, deeply and repeatedly during an outbreak, and sharing the WGS openly with the scientific community including product developers for characterization and diagnostic design. It is also imperative to perform deep sequencing in order to capture changing genomic profiles present in very minor proportions in the population within a host so that one can predict and adjust the signatures if the possibility of clonal expansion of a minor variant impacting the assay performance exists. 

It is critically important to assess the performance of assays in high consequence outbreaks against the species circulating in the outbreak. WGS of the circulating species can have a material impact on the spread of the disease, and on lives. While WGS is most desirable, more limited sequencing of the highly conserved regions may also produce a similar improvement. Sharing assay results with patient symptoms and outcome, via a multi-platform data repository that links these data set is needed. Ease of data entry and automated data communications are key requirements for such a system.

Currently deployed assays, even though designed to target regions of the genome believed to be conserved, have not been tested against new sequences available since their creation and may not have targeted the most highly conserved regions of the genome in the circulating viral population. We applied Bio*Velocity* to identify specific and robust signatures for each ebolavirus and then validated these results *in silico*. For EBOV, ten unique signatures were identified, none of which overlap with the currently used signatures including the 12 EBOV assays examined in this study indicating a gene-centric approach in the past assay design strategy rather than uniqueness. We have used a novel approach using the power of a large-memory kmer-based technique to rapidly design conserved and unique signatures. Bio*Velocity* could not run on normal cluster architectures with limited memory, or would take orders of magnitude longer if secondary storage were used for the kmer hash tables.

Tools such as Bio*Velocity* enable identification of unique and robust signatures for qPCR targets in minutes; however, to develop robust signatures with low false-negative rates, an adequate number of reference genomes are needed. The required size of the reference genome database is related to the genetic mutation rate and diversity of the population, and the tolerance for false-negative rates.

## Supplementary Materials

**Table S1:** Description of various Ebola assay targets and signature sequences used for PSET analysis.

**Table S2:** Source data used for generation of heat map shown in Figure 2.

**Table S3:** Bio*Velocity* analysis of ebolavirus genome sequences to develop new signatures.

**Table S4:** Description of various ebolavirus assay targets and sequences discovered using Bio*Velocity*.

**Table S5:** *In silico* validation of the uniqueness of species specific Bio*Velocity* signature sequences against other species.

**Document S5:** References used in Table S1.
