# Supplementary material for: Evaluation of Signature Erosion in Ebola Virus Due to Genomic Drift and Its Impact on the Performance of Diagnostic Assays
Source: Viruses. 2015 Jun 17;7(6):3130–54. doi: 10.3390/v7062763 (PMC4488730; doi:10.3390/v7062763)
Supplement: Supplementary file 1 [file viruses-07-02763-s001.zip › viruses-07-02763-supplementary/Supplementary_materials/Document_S5_References_Table_S1.docx]

**References used in Table S1**

1. Drosten, C.; Göttig, S.; Schilling, S.; Asper, M.; Panning, M.; Schmitz, H.; Günther, S. Rapid detection and quantification of rna of ebola and marburg viruses, lassa virus, crimean-congo hemorrhagic fever virus, rift valley fever virus, dengue virus, and yellow fever virus by real-time reverse transcription-pcr. *J Clin Microbiol* **2002**, *40*, 2323-2330.

2. Gibb, T.R.; Norwood, D.A.; Woollen, N.; Henchal, E.A. Development and evaluation of a fluorogenic 5' nuclease assay to detect and differentiate between ebola virus subtypes zaire and sudan. *J Clin Microbiol* **2001**, *39*, 4125-4130.

3. Gire, S.K.; Goba, A.; Andersen, K.G.; Sealfon, R.S.; Park, D.J.; Kanneh, L.; Jalloh, S.; Momoh, M.; Fullah, M.; Dudas, G.; *et al.* Genomic surveillance elucidates ebola virus origin and transmission during the 2014 outbreak. *Science* **2014**, *345*, 1369-1372.

4. Grard, G.; Biek, R.; Tamfum, J.J.; Fair, J.; Wolfe, N.; Formenty, P.; Paweska, J.; Leroy, E. Emergence of divergent zaire ebola virus strains in democratic republic of the congo in 2007 and 2008. *J Infect Dis* **2011**, *204 Suppl 3*, S776-784.

5. Kobinger, G.P.; Leung, A.; Neufeld, J.; Richardson, J.S.; Falzarano, D.; Smith, G.; Tierney, K.; Patel, A.; Weingartl, H.M. Replication, pathogenicity, shedding, and transmission of zaire ebolavirus in pigs. *J Infect Dis* **2011**, *204*, 200-208.

6. Morvan, J.M.; Deubel, V.; Gounon, P.; Nakouné, E.; Barrière, P.; Murri, S.; Perpète, O.; Selekon, B.; Coudrier, D.; Gautier-Hion, A.*, et al.* Identification of ebola virus sequences present as rna or dna in organs of terrestrial small mammals of the central african republic. *Microbes Infect* **1999**, *1*, 1193-1201.

7. Sanchez, A.; Ksiazek, T.G.; Rollin, P.E.; Miranda, M.E.; Trappier, S.G.; Khan, A.S.; Peters, C.J.; Nichol, S.T. Detection and molecular characterization of ebola viruses causing disease in human and nonhuman primates. *J Infect Dis* **1999**, *179 Suppl 1*, S164-169.

8. Towner, J.S.; Sealy, T.K.; Ksiazek, T.G.; Nichol, S.T. High-throughput molecular detection of hemorrhagic fever virus threats with applications for outbreak settings. *J Infect Dis* **2007**, *196 Suppl 2*, S205-212.

9. Trombley, A.R.; Wachter, L.; Garrison, J.; Buckley-Beason, V.A.; Jahrling, J.; Hensley, L.E.; Schoepp, R.J.; Norwood, D.A.; Goba, A.; Fair, J.N.*, et al.* Comprehensive panel of real-time taqman polymerase chain reaction assays for detection and absolute quantification of filoviruses, arenaviruses, and new world hantaviruses. *Am J Trop Med Hyg* **2010**, *82*, 954-960.

10. Weidmann, M.; Mühlberger, E.; Hufert, F.T. Rapid detection protocol for filoviruses. *J Clin Virol* **2004**, *30*, 94-99.
