# Supplementary material for: Evaluation of Signature Erosion in Ebola Virus Due to Genomic Drift and Its Impact on the Performance of Diagnostic Assays
Source: Viruses. 2015 Jun 17;7(6):3130–54. doi: 10.3390/v7062763 (PMC4488730; doi:10.3390/v7062763)
Supplement: Supplementary file 1 [file viruses-07-02763-s001.zip › viruses-07-02763-supplementary/Supplementary_materials/Table_S5_BV_Sig_Validation.docx]

**Table S4.** *In silico* validation of the uniqueness of species specific Bio*Velocity* signature sequences against other species

|  |  |  |  |  | Range of Percentage Identity  (Number of BLAST Hits) | | | | |
| --- | --- | --- | --- | --- | --- | --- | --- | --- | --- |
| Signature | Length | Start* | Stop* | Gene | Zaire | Tai Forest | Bundibugyo | Sudan | Reston |
| 1 | 86 | 3969 | 4054 | VP35 | 99-100  (137) | 77  (1) | 72  (5) | 69-70  (10) | 73-74  (8) |
| 2 | 86 | 6048 | 6133 | GP | 99-100  (156) | 76  (2) | 71  (5) | 60-62  (14) | 59-63  (12) |
| 3 | 146 | 6324 | 6469 | GP | 100  (156) | 78  (2) | 78  (5) | 77-79  (14) | 74-77  (12) |
| 4 | 77 | 7898 | 7974 | GP | 100  (156) | 71  (2) | 80  (5) | 69  (14) | 67-69  (12) |
| 5 | 123 | 8788 | 8910 | VP30 | 100  (136) | 72  (1) | 74  (5) | 69-70  (10) | 64-66  (8) |
| 6 | 95 | 9268 | 9362 | VP30 | 99-100  (136) | 62  (1) | 69  (5) | 50-54  (10) | 46-48  (8) |
| 7 | 80 | 10663 | 10742 | VP24 | 99-100  (138) | 73  (1) | 76-77  (5) | 69-70  (10) | 70-73  (8) |
| 8 | 80 | 13687 | 13766 | L | 100  (136) | 88  (1) | 84  (5) | 75-76  (11) | 76-81  (8) |
| 9 | 92 | 13927 | 14018 | L | 100  (136) | 88  (1) | 83  (5) | 77-81  (11) | 81-85  (8) |
| 10 | 88 | 15511 | 15598 | L | 99-100  (136) | 71  (1) | 68-69  (5) | 67-68  (11) | 75-76  (8) |

|  |  |  |  |  | Range of Percentage Identity  (Number of BLAST hits) | | | | |
| --- | --- | --- | --- | --- | --- | --- | --- | --- | --- |
| Signature | Length | Start* | Stop* | Gene | Zaire | Tai Forest | Bundibugyo | Sudan | Reston |
| 11 | 77 | 428 | 504 | NP* | 30-31  (137) | 35  (1) | 42-43  (5) | 100  (11) | 44-45  (8) |
| 12 | 80 | 983 | 1062 | NP | 86  (129) | 78  (1) | 81-82  (5) | 100  (10) | 86-89  (8) |
| 13 | 83 | 1820 | 1902 | NP | 44-48  (129) | 54  (1) | 54-55  (5) | 100  (10) | 58-61  (8) |
| 14 | 112 | 4655 | 4766 | VP40 | 74-75  (139) | 72  (1) | 74  (5) | 100  (10) | 74-76  (8) |
| 15 | 120 | 4874 | 4993 | VP40 | 80-82  (139) | 81  (1) | 76-77  (5) | 100  (10) | 74-79  (8) |
| 16 | 107 | 6817 | 6923 | GP | 64-66  (156) | 68  (2) | 68  (5) | 100  (14) | 72-75  (12) |
| 17 | 86 | 7527 | 7612 | GP | 65-69  (156) | 64  (2) | 69  (5) | 100  (14) | 57-60  (12) |
| 18 | 91 | 9821 | 9911 | VP24* | 34-35  (136) | 28  (1) | 24  (5) | 100  (10) | 23-24  (8) |
| 19 | 76 | 10288 | 10363 | VP24* | 68-71  (136) | 62  (1) | 70-71  (5) | 100  (10) | 64-66  (8) |
| 20 | 89 | 12435 | 12523 | L | 71-72  (136) | 66  (1) | 71  (5) | 100  (11) | 72-73  (8) |
| 21 | 92 | 13455 | 13546 | L | 75-76  (136) | 71  (1) | 68  (5) | 100  (11) | 76-77  (8) |
| 22 | 80 | 14932 | 15011 | L | 65-68  (136) | 59  (1) | 55  (5) | 100  (11) | 56-58  (8) |
| 23 | 92 | 15702 | 15793 | L | 62-65  (136) | 64  (1) | 70-72  (5) | 100  (11) | 70-71  (8) |
| 24 | 80 | 17049 | 17128 | L | 72-74  (136) | 68  (1) | 65-66  (5) | 100  (11) | 66-68  (8) |
| 25 | 176 | 17784 | 17959 | L | 66  (136) | 70  (1) | 69  (5) | 100  (11) | 66-67  (8) |

*Denotes signature is in leader sequence of gene

|  |  |  |  |  | Range of Percentage Identity  (Number of BLAST hits) | | | | |
| --- | --- | --- | --- | --- | --- | --- | --- | --- | --- |
| Signature | Length | Start* | Stop* | Gene | Zaire | Tai Forest | Bundibugyo | Sudan | Reston |
| 26 | 101 | 1388 | 1488 | NP | 81-83  (129) | 76  (1) | 76-77  (5) | 76-78  (10) | 100  (8) |
| 27 | 82 | 2505 | 2586 | NP | 71-72  (129) | 68  (1) | 72-73  (5) | 67-69  (10) | 100  (8) |
| 28 | 92 | 3434 | 3525 | VP35 | 63-64  (137) | 73  (1) | 54-55  (5) | 67-68  (10) | 100  (8) |
| 29 | 83 | 3800 | 3882 | VP35 | 83  (137) | 76  (1) | 77  (5) | 77-81  (10) | 100  (8) |
| 30 | 80 | 4614 | 4693 | VP40 | 62-69  (139) | 69  (1) | 70  (5) | 77-81  (10) | 100  (8) |
| 31 | 86 | 8757 | 8842 | VP30 | 63-64  (136) | 62  (1) | 58  (5) | 56-57  (10) | 100  (8) |
| 32 | 124 | 8924 | 9047 | VP30 | 64-66  (136) | 64  (1) | 68  (5) | 60-61  (10) | 100  (8) |
| 33 | 86 | 10645 | 10730 | VP24 | 69-70  (138) | 62  (1) | 60-64  (5) | 62-67  (10) | 100  (8) |
| 34 | 88 | 12036 | 12123 | L | 76  (136) | 76  (1) | 80  (5) | 69-70  (11) | 100  (8) |
| 35 | 80 | 12330 | 12409 | L | 54-56  (136) | 59  (1) | 53-55  (5) | 54-58  (11) | 100  (8) |
| 36 | 89 | 12876 | 12964 | L | 74-75  (136) | 79  (1) | 75  (5) | 78  (11) | 100  (8) |
| 37 | 104 | 13521 | 13624 | L | 78-80  (136) | 71  (1) | 74  (5) | 76-78  (11) | 100  (8) |
| 38 | 98 | 13821 | 13918 | L | 78-80  (136) | 72  (1) | 78  (5) | 74-76  (11) | 100  (8) |
| 39 | 110 | 14208 | 14317 | L | 65-68  (136) | 78  (1) | 74  (5) | 73-75  (11) | 100  (8) |
| 40 | 89 | 14763 | 14851 | L | 70-72  (136) | 74  (1) | 75-76  (5) | 68-73  (11) | 100  (8) |
| 41 | 101 | 15027 | 15127 | L | 70-71  (136) | 71  (1) | 68  (5) | 71  (11) | 100  (8) |
| 42 | 98 | 17511 | 17608 | L | 60-62  (136) | 63  (1) | 62-64  (5) | 63-65  (11) | 100  (8) |
